# Supplementary material for: Genetic Instability among Hitnü People Living in Colombian Crude-Oil Exploitation Areas
Source: Int J Environ Res Public Health. 2022 Sep 6;19(18):11189. doi: 10.3390/ijerph191811189 (PMC9517229; doi:10.3390/ijerph191811189)
Supplement: Supplementary file 1 [file ijerph-19-11189-s001.zip › ijerph-1848859-supplementary.pdf]

Supplementary Materials

Table S1. Comparison of CBMN-cyt assay parameters among non-exposed and exposed Hitnü indigenous.

Parameter and variables	Non-exposed			Exposed			
	n	Mean ± SD	P_{50} (P_{25}-P_{75})	n	Mean ± SD	P_{50} (P_{25}-P_{75})	p-value
**DNA damage**							
**MN**	40	3.72 ± 2.94	3.0 (2.00 – 5.00)	58	5.98 ± 6.35	4.0 (200 – 8.75)	0.22
Women	18	3.11 ± 2.65	3.0 (1.25 – 4.00)	29	6.24 ± 6.08	5.0 (2.00 – 8.00)	0.06
Men	22	4.22 ± 3.13	3.5 (2.25 – 5.75)	29	5.72 ± 6.70	3.0 (1.00 – 9.00)	0.93
**NPB**	40	0.85 ± 1.57	0.0 (0.00 – 1.00)	58	1.56 ± 2.02	**1.0 (0.00 – 2.00)**	**0.03** [b]
Women	18	0.88 ± 1.49	0.0 (0.00 – 1.75)	29	1.79 ± 2.32	1.0 (0.00 – 3.00)	0.15
Men	22	0.81 ± 1.68	0.0 (0.00 – 1.00)	29	1.34 ± 1.69	1.0 (0.00 – 2.00)	0.11
**NBUD**	40	0.30 ± 0.72	0.0 (0.00 – 0.00)	58	0.15 ± 0.48	0.0 (0.00 – 0.00)	0.30 [b]
Women	18	0.33 ± 0.68	0.0 (0.00 – 0.00)	29	**0.03 ± 0.18**	**0.0 (0.00 – 0.00)**	**0.04** [a]
Men	22	0.27 ± 0.76	0.0 (0.00 – 0.00)	29	0.27 ± 0.64	0.0 (0.00 – 0.00)	0.78
**Cytostatic and Cell death**							
**APOP**	40	2.27 ± 3.31	1.0 (0.00 – 3.25)	58	5.77 ± 8.70	1.0 (0.00 – 9.00)	0.22
Women	18	1.72 ± 2.76	0.0 (0.00 – 2.75)	29	5.24 ± 8.42	1.0 (0.00 – 7.00)	0.21
Men	22	2.72 ± 3.70	1.5 (0.00 – 3.75)	29	6.31 ± 9.09	1.0 (0.00 – 9.00)	0.54
**NECRO**	40	1.22 ± 2.54	1.0 (0.00 – 1.00)	58	1.39 ± 2.74	1.0 (0.00 – 1.00)	0.84
Women	18	0.61 ± 1.91	0.0 (0.00 – 0.00)	29	1.89 ± 3.37	0.0 (0.00 – 1.00)	0.12
Men	22	1.72 ± 2.91	0.0 (0.00 – 2.50)	29	0.89 ± 1.85	1.0 (0.00 – 1.00)	0.25
**NDI**	40	2.07 ± 0.21	2.07 (1.99 – 2.18)	58	2.01 ± 0.26	2.0 (1.86 – 2.12)	0.10
Women	18	2.04 ± 0.19	2.00 (2.00 – 2.16)	29	2.06 ± 0.27	2.0 (1.88 – 2.18)	0.65
Men	22	2.09 ± 0.23	2.09 (1.97 – 2.22)	29	1.95 ± 0.23	1.9 (1.83 – 2.11)	0.07

Bold for statistically significant values.

SD: Standard deviation.

[a] significant association in comparison to men with the same exposure status.

[b] significant association in comparison to non-exposed individuals

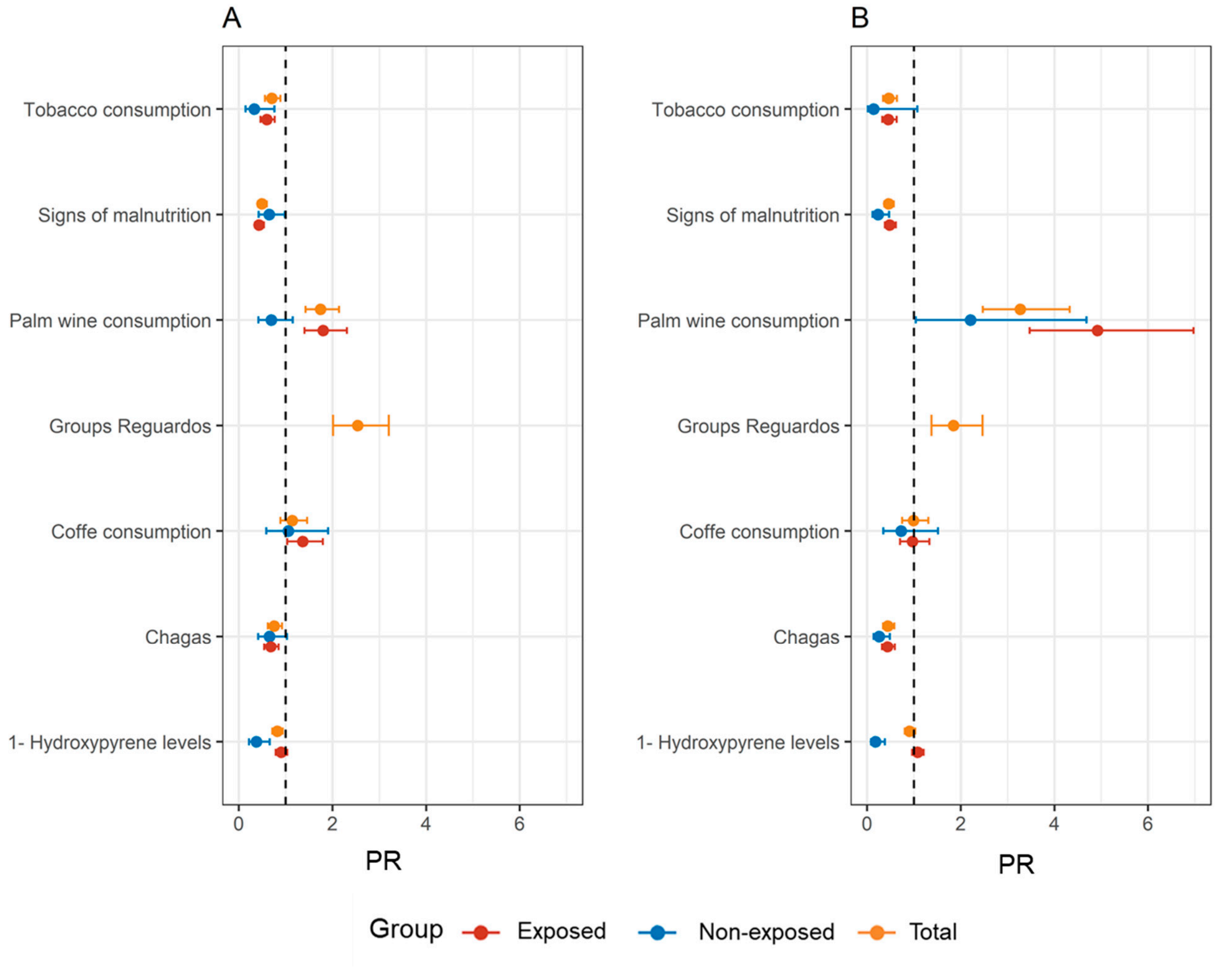

Figure S1. IRR (proxy or PR) for Apoptotic cells frequency based on a Poisson regression model by exposure groups. A) unadjusted and B) adjusted for covariates.

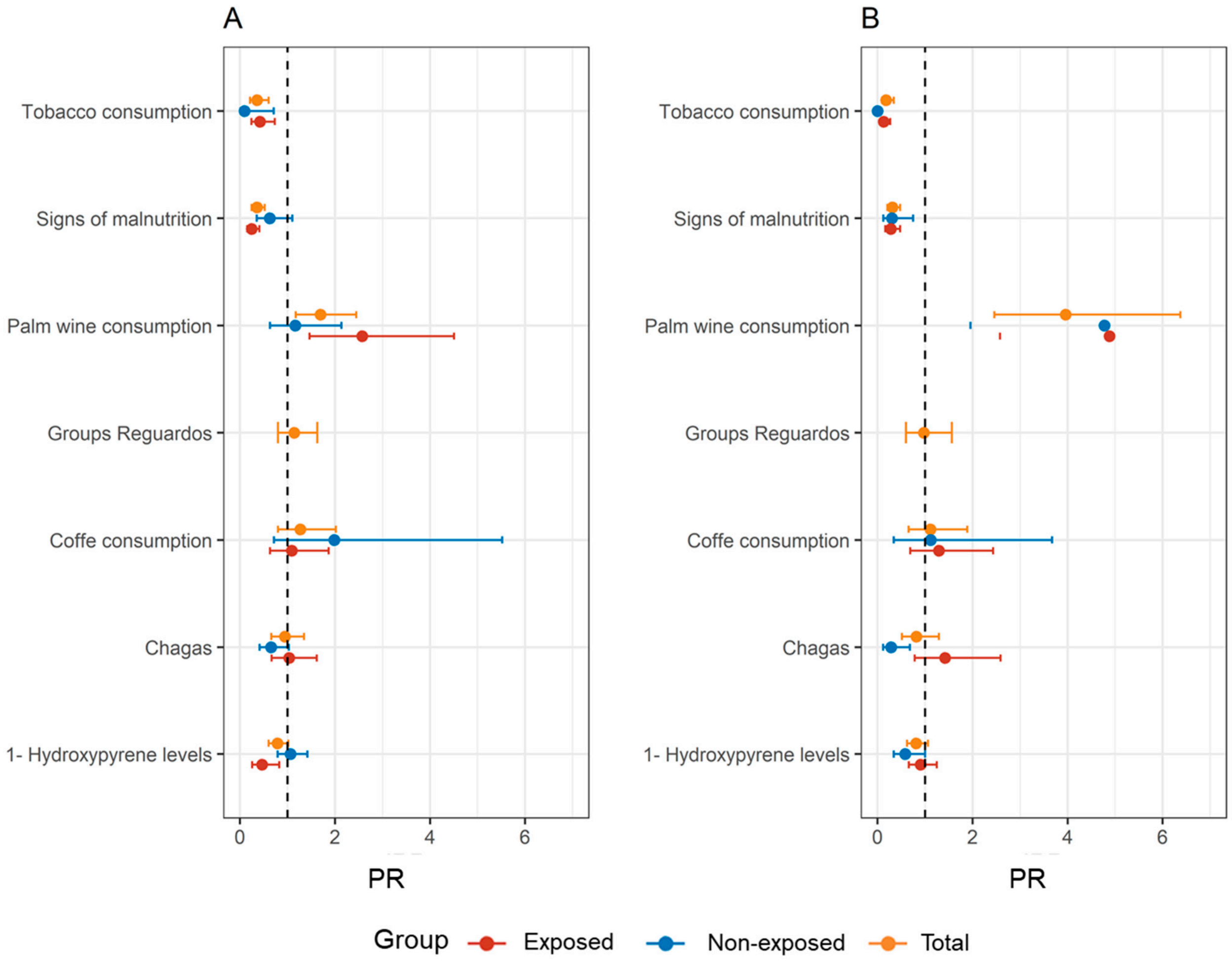

Figure S2. IRR (proxy or PR) for Necrotic cells frequency based on a Poisson regression model by exposure groups. A) unadjusted and B) adjusted for covariates.
